# Genomic Profiling Reveals Novel Predictive Biomarkers for Chemo-Radiotherapy Efficacy and Thoracic Toxicity in Non-Small-Cell Lung Cancer

**DOI:** 10.3389/fonc.2022.928605

**Published:** 2022-07-14

**Authors:** Kewen He, Shaotong Zhang, Jiaohui Pang, Jiani C. Yin, Dianbin Mu, Jun Wang, Hong Ge, Jie Ma, Zhe Yang, Xiaoli Zheng, Lihua Dong, Junli Zhang, Pengyu Chang, Li Li, Shanshan Tang, Hua Bao, Xue Wu, Xiaonan Wang, Yang Shao, Jinming Yu, Shuanghu Yuan

**Affiliations:** ^1^ Department of Radiation Oncology, Shandong University Cancer Center, Shandong Cancer Hospital and Institute, Shandong First Medical University and Shandong Academy of Medical Sciences, Jinan, China; ^2^ Department of Ultrasound, Jinan Central Hospital Affiliated to Shandong First Medical University, Jinan, China; ^3^ Geneseeq Research Institute, Nanjing Geneseeq Technology Inc., Nanjing, China; ^4^ Department of Pathology, Shandong Cancer Hospital and Institute, Shandong First Medical University and Shandong Academy of Medical Sciences, Jinan, China; ^5^ Department of Radiation Oncology, Fourth Hospital of Hebei Medical University, Shijiazhuang, China; ^6^ Department of Radiation Oncology, The Affiliated Cancer Hospital of Zhengzhou University, Zhengzhou, China; ^7^ Department of Pathology, The Affiliated Cancer Hospital of Zhengzhou University, Zhengzhou, China; ^8^ Department of Radiation Oncology, Shandong Provincial Hospital, Jinan, China; ^9^ Department of Radiation Oncology & Therapy, Jilin Provincial Key Laboratory of Radiation Oncology & Therapy, The First Hospital of Jilin University, Jilin, China; ^10^ School of Public Health, Nanjing Medical University, Nanjing, China

**Keywords:** non-small cell lung cancer, radiotherapy, radiation sensitivity, biomarker, genetic variation, prognosis, chemo-radiotherapy, radiotherapy-associated toxicity

## Abstract

Chemo-radiotherapy (CRT) remains the main treatment modality for non-small-cell lung cancer (NSCLC). However, its clinical efficacy is largely limited by individual variations in radio-sensitivity and radiotherapy-associated toxicity. There is an urgent need to identify genetic determinants that can explain patients’ likelihood to develop recurrence and radiotherapy-associated toxicity following CRT. In this study, we performed comprehensive genomic profiling, using a 474-cancer- and radiotherapy-related gene panel, on pretreatment biopsy samples from patients with unresectable stage III NSCLCs who underwent definitive CRT. Patients’ baseline clinical characteristics and genomic features, including tumor genetic, genomic and molecular pathway alterations, as well as single nucleotide polymorphisms (SNPs), were correlated with progression-free survival (PFS), overall survival (OS), and radiotherapy-associated pneumonitis and/or esophagitis development after CRT. A total of 122 patients were enrolled between 2014 and 2019, with 84 (69%) squamous cell carcinomas and 38 (31%) adenocarcinomas. Genetic analysis confirmed the association between the KEAP1-NRF2 pathway gene alterations and unfavorable survival outcome, and revealed alterations in *FGFR* family genes, *MET*, *PTEN*, and *NOTCH2* as potential novel and independent risk factors of poor post-CRT survival. Combined analysis of such alterations led to improved stratification of the risk populations. In addition, patients with *EGFR* activating mutations or any oncogenic driver mutations exhibited improved OS. On the other hand, we also identified genetic markers in relation to radiotherapy-associated thoracic toxicity. SNPs in the DNA repair-associated *XRCC5* (rs3835) and *XRCC1* (rs25487) were associated with an increased risk of high-grade esophagitis and pneumonitis respectively. *MTHFR* (rs1801133) and *NQO1* (rs1800566) were additional risk alleles related to higher susceptibility to pneumonitis and esophagitis overall. Moreover, through their roles in genome integrity and replicative fidelity, somatic alterations in *ZNF217* and *POLD1* might also serve as risk predictors of high-grade pneumonitis and esophagitis. Taken together, leveraging targeted next-generating sequencing, we identified a set of novel clinically applicable biomarkers that might enable prediction of survival outcomes and risk of radiotherapy-associated thoracic toxicities. Our findings highlight the value of pre-treatment genetic testing to better inform CRT outcomes and clinical actions in stage III unresectable NSCLCs.

## Introduction

Lung cancer is the leading cause of cancer-related deaths worldwide and in China, among which approximately 85% of patients have non-small cell lung cancers (NSCLCs) (1, 2). NSCLC is sub-categorized based on histological features into mainly adenocarcinoma (ADC) and squamous cell carcinoma (SCC) (1). Over the past two decades, many therapeutic advances have been made given our deepened understanding of lung cancer etiology. The identification of actionable molecular targets has revolutionized the management of NSCLC, with targeted therapies demonstrating remarkable clinical benefits in patients carrying specific driver mutations (1). Nevertheless, the majority of lung cancer patients still require radiotherapy for cure or palliative care. In particular, for NSCLC patients with unresectable locally advanced tumors, especially SCC, the combination of chemotherapy and thoracic radiation, given either concurrently or sequentially, remains the standard of care (3).

Radiotherapy, together with the radio-sensitizing effect of chemotherapy, results in enhanced anti-tumor efficacy, although at the expense of significant normal tissue toxicity. Radiotherapy-induced lung injury (known as radiation pneumonitis in an early phase and pulmonary fibrosis in the late phase), as well as esophagitis are common adverse events following thoracic radiation (4, 5). There are considerable variations between patients in their likelihood to develop severe adverse events following a given dose of radiation, which consequently, limits the maximum dose that can be administered to the majority (6). Similarly, there are substantial differences in individual response to chemo-radiotherapy (CRT) and the risk of resistance development. It has been long recognized that genetic variations between individuals or tumors are major contributors to the differences in radio-sensitivity and risks of developing radiotherapy-associated toxicity, and thus, must be taken into consideration for personalized radiotherapy dose-prescription.

Our understanding of differential response to radiotherapy begins with the discovery of several genetic syndromes caused by mutations in the DNA repair pathways, which can lead to life-threatening radiotherapy toxicity (7, 8). Subsequently, a number of other molecular processes, such as scavenging of reactive oxygen species (ROS), apoptosis, proliferation and inflammatory response, have been implicated in the development of radiation-induced toxicity (9–12). While multiple approaches, including candidate gene approach and genome-wide association studies, were undertaken to identify genetic variants that might explain the differences in individual response to radiotherapy, no robust biomarkers with convincing clinical applicability have been identified (13). In addition, a lung tissue-specific, comprehensive analysis for personalized radiotherapy is still lacking. Here, taking advantage of next-generation sequencing (NGS) technology, we performed comprehensive genomic profiling on 474 cancer- and radio-sensitivity-related genes of the tumor biopsies from 122 unresectable stage III NSCLC patients prior to radiation therapy, and identified a set of promising biomarkers for predicting radiation survival and toxicity, which may prove beneficial for guiding clinical treatment decision-making.

## Material and Methods

### Patient Enrollment

The patients with NSCLC in the study were treated with CRT at the multiple centers between October 2014 and March 2019. Eligible patients for this study were determined based on the following criteria: histological diagnosis of unresectable stage IIIA-C NSCLC based on the tumor, node and metastasis (TNM) staging system without severe pleural or pericardial effusion, age older than 18 years, adequate lung, bone marrow, renal, hepatic, and cardiac function, and no history of systemic treatment or radiotherapy for thoracic cancers. The study was approved by the Ethical Review Board of the Oncology Center of Shandong Provincial Hospital, and all patients provided written informed consent.

### Treatment and Assessments

All patients in this study received standard definitive CRT (dCRT). A median of five cycles of cisplatin- or paclitaxel-based chemotherapy were given concurrently or sequentially with radiotherapy. The choice of chemotherapy regimen was left to the investigator’s discretion. Three-dimensional conformal radiation therapy (3D-CRT) or intensity-modulated radiation therapy (IMRT) was administered at a total dose of 50-70 Gy.

The follow-up of all patients was conducted 1 month after radiotherapy, and then every 3 months during the first year. After that, the patients were followed up every 3-6 months. Radiotherapy-associated thoracic toxicities were graded according to the toxicity criteria of the Radiation Therapy Oncology Group (RTOG) and the European Organization for Research and Treatment of Cancer (EORTC) (14). For toxicity criteria of pneumonitis, grade 1 includes mild symptoms of dry cough or dyspnea on exertion; grade 2 includes persistent cough requiring narcotic or antitussive agents, or dyspnea with minimal effort but not at rest; grade 3 includes severe cough unresponsive to narcotic antitussive agent or dyspnea at rest, clinical or radiological evidence of acute pneumonitis, or requirement of intermittent oxygen or steroids; and grade 4 includes severe respiratory insufficiency or continuous oxygen or assisted ventilation. For esophagitis, grade 1 includes mild dysphagia or odynophagia, requirement of topical anesthetic, non-narcotic analgesics, or soft diet; grade 2 includes moderate dysphagia or odynophagia, requirement of narcotic analgesics, puree or liquid diet; grade 3 includes severe dysphagia or odynophagia with dehydration or weight loss >15% from pretreatment baseline, requirement of nasogastric feeding tube, intravenous fluids, or hyperalimentation; grade 4 includes complete obstruction, ulceration, perforation, or fistula. Treatment responses were assessed using CT imaging at each follow-up and compared to the images at baseline or from the last follow-up and were evaluated according to the Response Evaluation Criteria in Solid Tumors (RECIST), version 1.1. Progression-free survival (PFS) was defined as the time from the beginning of treatment to disease progression. Patients who had not progressed were censored at the date of their last scan. Overall survival (OS) was defined from the beginning of treatment to the time of death from any cause or the last follow-up.

### DNA Extraction and Library Preparation

All tumor samples were formalin-fixed paraffin-embedded (FFPE, 10 µm) and were obtained from original biopsies prior to any treatment. At least 10% tumor content of all samples, as determined by pathologists, was required. NGS was performed in a CLIA-certified and CAP-accredited laboratory (Nanjing Geneseeq Technology Inc., Nanjing, China). Genomic DNA was extracted from de-paraffinized FFPE sections using QIAamp DNA FFPE Tissue Kit (Qiagen) according to the manufacturer’s instructions. Quantity and quality of DNA were assessed using Qubit 3.0 fluorometer and Nanodrop 2000 (ThermoFisher), respectively. DNA was fragmented into 350 bp using the Covaris M220 sonication system and purified with Agencourt AMPure XP beads (Beckman Coulter).

DNA (50 ng) libraries were prepared with KAPA hyper library preparation kit (KAPA Biosystems). Libraries with different indices were pooled for targeted enrichment with IDT xGen Lockdown Reagents and a customized enrichment panel (IDT) covering 474 cancer-related genes with whole-exon coverage, including those that have been implicated in radiotherapy response and/or radiotherapy-associated adverse effects (Radiotron^®^, Nanjing Geneseeq Technology Inc., Nanjing; **Supplementary Table 1**). Libraries were captured with Dynabeads M-270 (Life Technologies) and xGen Lockdown hybridization and wash kit (IDT). The captured library was further on-beads PCR amplified with Illumina p5 (5’ AAT GAT ACG GCG ACC GA 3’) and p7 (5’ CAA GCA GAA GAC GGC ATA CGA GAT 3’) primers in KAPA Hifi HotStart ReadyMix (KAPA Biosystems) and purified with Agencourt AMPure XP beads. Sequencing libraries were sized on the Agilent Bioanalyzer 2100 (Agilent Technologies) and their concentrations analyzed by qPCR with KAPA Library Quantification kit (KAPA Biosystems). The final libraries were sequenced on an Illumina Hiseq 4000 platform to a mean coverage depth of ~350.

### Sequencing Data Analysis

NGS read preprocessing, including quality control of FASTQ files and removing leading/trailing low quality (quality reading below 15) or N bases, was conducted with Trimmomatic (15). Qualified pair-end reads were aligned to the reference human genome hg19 with Burrows-Wheeler Aligner (v0.7.12) (16). PCR deduplication was performed using Picard and local realignment around indels and base quality score recalibration was performed using GATK3. Samples with mean dedup depth of less than 30X were also removed. Cross-sample contamination was quantified by using ContEst (Broad Institute). Single nucleotide polymorphisms (SNPs) were identified if present in >1% population frequency in the 1000g, genomAD, or ExAC databases. Somatic single nucleotide variants (SNVs) and indels were identified using VarScan2 (17) with the following parameters: i) minimum read depth=20; ii) minimum variant supporting reads=5, mapped to both strands; iii) minimum base quality=15; iv) strand bias no greater than 10%. Somatic variants were further filtered through an internally collected list of recurrent sequencing errors and if present in >1% population frequency in the 1000g, genomAD or ExAC database. Copy number variations (CNVs) were detected using CNVkit (18). CNV gain and loss were identified if depth ratio were above 1.6 or below 0.6, respectively. Final list of mutations was annotated using vcf2maf (call VEP for annotation). Panel tumor mutational burden (TMB) was counted by summing all base substitutions and indels in the coding region of targeted genes, including synonymous alterations to reduce sampling noise and excluding known driver mutations as they are over-represented in the Panel, as previously described (19).

### Statistical Analysis

For comparisons of proportion between groups, Fisher’s exact tests were performed. For non-normally distributed data, such as TMB, differences between two groups were evaluated with the non-parametric Mann-Whitney/Wilcoxon rank-sum test. For survival analyses, Kaplan-Meier curves were estimated using the log-rank test, and hazard ratios (HRs) for PFS and OS were calculated by Cox proportional hazards model. Multivariable survival analysis was performed using the Cox regression model. A two-sided P value of less than 0.05 was considered to be statistically significant unless otherwise indicated. All statistical analyses were done in R (v.3.5.2).

## Results

### Patient Overview

We retrospectively performed analyses on 122 patients with unresectable stage III NSCLC, who underwent dCRT. Patients’ baseline characteristics were summarized in **Table 1**. Median age of the study cohort was 62 years. Histological subtypes included 84 SCC (68.9%) and 38 ADC (31.1%). Consistent with a higher proportion of SCC patients, there were more male patients (87.7%) and former smokers (74.6%) in the study cohort. 51.6% (63/122) patients received concurrent dCRT and the remaining (48.4%) received sequential dCRT. At data cutoff, the median follow-up time was 30.1 months. Median PFS and OS of the study cohort were 11.4 and 34.6 months, respectively (**Supplementary Figure 1**). A total of 51 (41.8%) patients developed grade 2 or higher toxicity, with 39 patients developed grade 2 or higher pneumonitis and 16 cases of esophagitis.

**Table 1:** Clinical characteristics of the patients.

Characteristics	No. of patients (%)
**Sex**	
Male	107 (87.7%)
Female	15 (12.3%)
**Age**	
<65	73 (59.8%)
≥65	49 (40.2%)
**Median age (range)**	62 (33–84)
**Histological subtypes**	
Adenocarcinoma	38 (31.1%)
Squamous cell carcinoma	84 (68.9%)
**Smoking history**	
Former smokers	91 (74.6%)
Never smokers	31 (25.4%)
**Treatment regimen**	
SCRT	59 (48.4%)
CCRT	63 (51.6%)
**Radiation type**	
3DCRT	30 (24.6%)
IMRT	92 (75.4%)
Radiation dose	
<60 Gy	28 (23.0%)
=60 Gy	66 (54.0%)
>60 Gy	28 (23.0%)
**RT-related pneumonitis**	
Grade 0-1	83 (68.0%)
Grade 2	18 (14.8%)
Grade 3	19 (15.6%)
Grade 4	2 (1.6%)
**RT-related esophagitis**	
Grade 0-1	106 (86.9%)
Grade 2	8 (6.55%)
Grade 3	8 (6.55%)
Grade 4	0 (0%)

SCRT, sequential chemoradiotherapy; CCRT, concurrent chemoradiotherapy

**Figure 1:**
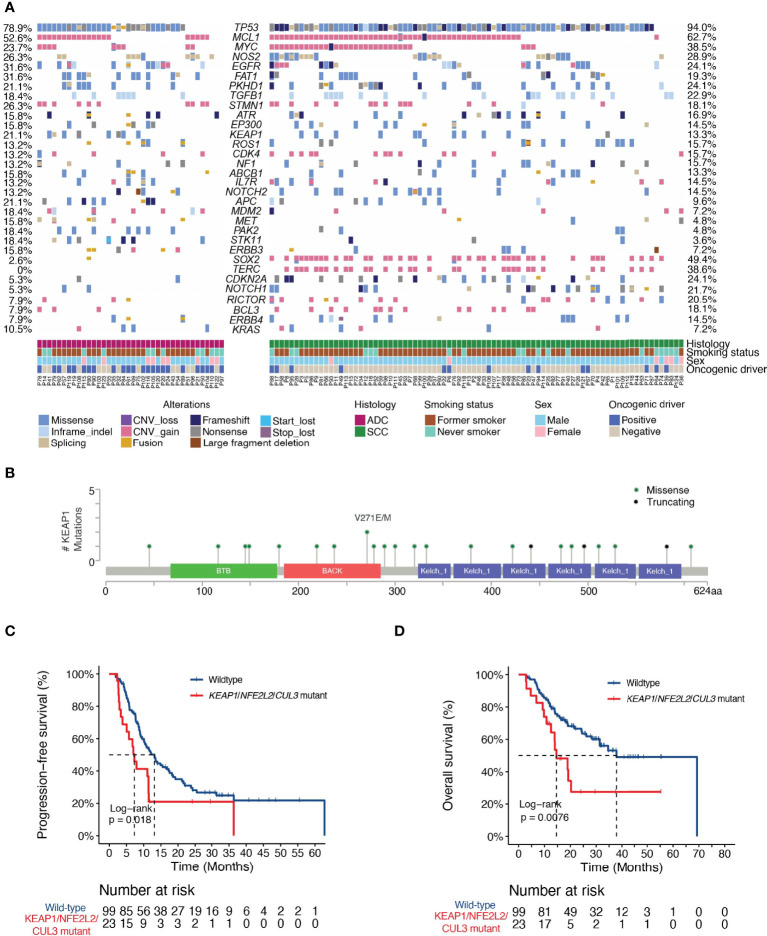
Landscape of genetic variations and the associations of dCRT survival outcomes with the KEAP1-NRF2 pathway. **(A)** The distribution of various genetic variations in each patient was shown. Clinical characteristics of each patient were shown at the bottom. ADC, adenocarcinoma; SCC, squamous cell carcinoma. **(B)** Lollipop plot showing the distribution of *KEAP1* mutations in the study cohort. **(C, D)** Kaplan-Meier estimates of **(C)** PFS and **(D)** OS in the full analysis set comparing patients with and without KEAP1-NRF2 pathway gene mutations. HR denotes hazard ratio; CI denotes confidence interval. Tick marks indicate censored data.

Genomic profiling on baseline tissues using a targeted NGS panel covering 474 cancer- and radiotherapy-associated genes (**Supplementary Table 1**) revealed the mutational landscape of the cohort (**Figure 1A**). The most frequently altered genes were *TP53* (ADC, 78.9%; SCC, 94.0%), *MCL1* (ADC, 52.6%; SCC, 62.7%), *MYC* (ADC, 23.7%; SCC, 38.5%), *NOS2* (ADC, 26.3%; SCC, 28.9%) and *EGFR* (ADC, 31.6%; SCC 24.1%). Notably, we observed a high frequency of *TP53* and a low frequency of *EGFR* mutations in our ADC patients, which was consistent with an enrichment of former smokers in our study. The median TMB of the cohort is 13.4 mutations/Mb, with no significant difference observed between ADC (12.4 mutations/Mb) and SCC (13.9 mutations/Mb; *P*=0.68).

### Predictive Markers for Survival Outcome Following CRT

First, we examined potential associations between clinical characteristics and survival outcome following dCRT. No significant difference in survival outcomes was observed comparing patients with different histological subtypes (PFS, *P*=0.27; OS, *P*=0.76). Patients treated with concurrent and sequential dCRT also had similar PFS outcome (*P*=0.56, **Supplementary Figure 2A**), albeit a small trend towards increased OS in patients treated with concurrent dCRT (HR [95% CI] =0.63 [0.37-1.1], *P*=0.10, **Supplementary Figure 2B**). Patients treated with 3D-conformal RT had lower risk of progression compared with those treated with intensity-modulated RT (HR [95% CI] =0.53 [0.31-0.90], *P*=0.01, **Supplementary Figure 2C**), which did not translate into an OS difference (*P*=0.92, **Supplementary Figure 2D**). Patients with smoking histories had worse outcome compared with never smokers (PFS, HR [95% CI] =1.95 [1.15-3.32], *P*=0.01; OS, HR [95% CI] =1.58 [0.79-3.16], *P*=0.19, **Supplementary Figures 2E, F**). A higher overall dose was associated with a trend towards improved PFS and prolonged OS (PFS, HR [95% CI] =0.73 [0.45-1.18], *P*=0.2; OS, HR [95% CI] =0.56 [0.31-1.0], *P*=0.05, **Supplementary Figures 2G, H**).

Next, we explored the associations between individual genomic alterations (non-synonymous alterations that occur in at least 5% of the cohort) and dCRT survival outcome. We identified 19 patients with mutations in Kelch Like ECH Associated Protein 1 (*KEAP1*, **Figures 1A, B**). KEAP1 is an E3 ubiquitin ligase that functions as a sensor for oxidative stress and negatively regulates NRF2, a transcription factor upstream of cytoprotective and antioxidant genes, in the absence of stress (20). Of the 19 *KEAP1*-mutant patients, three carried nonsense mutations and the rest carried missense mutations that were all except three predicted to be deleterious or potentially damaging to protein function by SIFT or PolyPhen. Specifically, we detected three mutations in the BTB domain and four in the intervening BACK (BTB and C-terminal Kelch) domain, both of which mediate its interaction with Cullin 3 (Cul3) for protein ubiquitination, as well as ten mutations located in the Kelch repeat domains that mediates interaction with NRF2 (**Figure 1B**). *KEAP1* mutations have been reported to correlate with an increased rate of local recurrence in NSCLC patients treated with radiotherapy (21). Indeed, our data independently showed that patients with *KEAP1* mutations had shorter median PFS (6.7 months vs. 12.2 months; HR [95% CI] = 2.17 [1.24-3.81], *P* = 0.006, **Supplementary Figure 3A**) and OS (18.8 vs. 37.8 months; HR [95% CI] =2.37 [1.23-4.55], *P*=0.008, **Supplementary Figure 3B**) compared with those with the wild-type gene. Considering patients with deleterious mutations in genes in the KEAP1-NRF2 pathway (*KEAP1*, *NFE2L2* or *CUL3*) showed a consistent increase in the risk of disease progression (HR [95% CI] =1.86 [1.1-3.15], *P*=0.02, **Figure 1C**), as well as decreased OS (HR=2.27 [1.22-4.23], *P*=0.008, **Figure 1D**).

**Figure 2:**
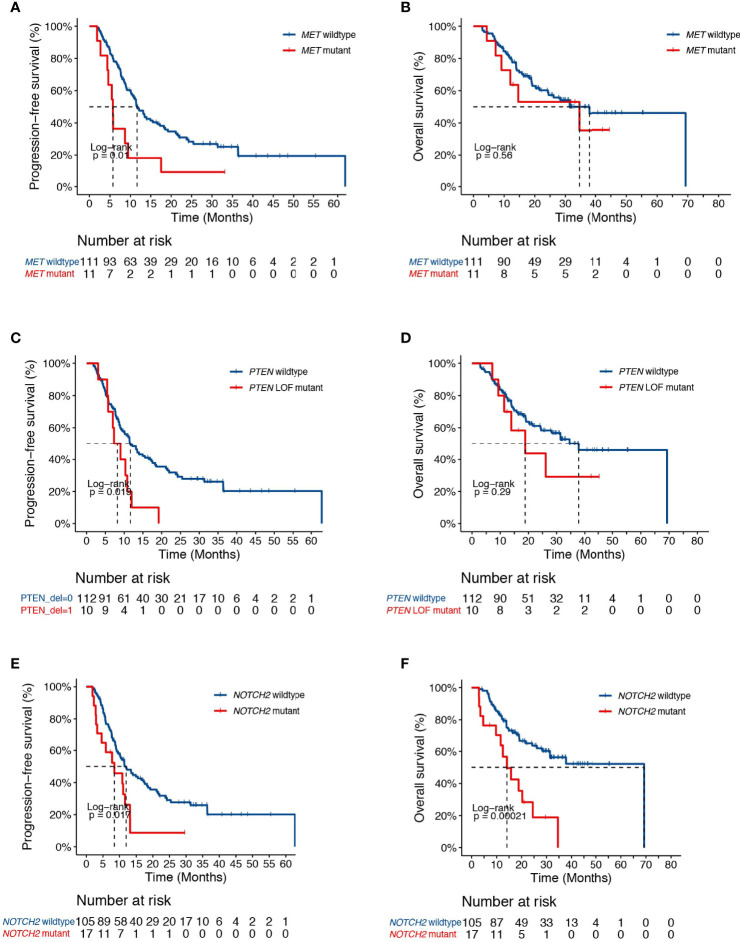
Clinical associates of dCRT survival outcomes in NSCLC. **(A, B)** Kaplan-Meier estimates of **(A)** PFS and **(B)** OS in the full analysis set comparing patients with and without *MET* alterations. **(C, D)** Kaplan-Meier estimates of **(C)** PFS and **(D)** OS in the full analysis set comparing patients with and without *PTEN* deleterious mutations. **(E, F)** Kaplan-Meier estimates of **(E)** PFS or **(F)** OS in the full analysis set comparing patients with and without *NOTCH2* alterations.

Univariate analysis revealed additional associations of survival outcome following dCRT with variations in several key genes that play important roles in lung cancer initiation and progression. The *MET* oncogene, which encodes a receptor tyrosine kinase, has become an important target for the treatment of NSCLC. We identified 11 patients with *MET* alterations, of which one had *MET* amplification, four carried exon 14 skipping mutations, three had *MET* fusions, and one patient with both amplification and an exon 14 skipping mutation (**Figure 1A**). The presence of *MET* alterations had a negative impact on disease progression, with shorter PFS than those with the wild-type gene (HR [95% CI] =2.33 [1.2-4.52], *P*=0.01, **Figure 2A**). No significant difference in OS was found comparing patients with *MET* alterations and those with the wild-type gene (HR [95% CI] =1.29 [0.55-3.03], *P*=0.56, **Figure 2B**). *PTEN* is an important tumor suppressor gene in lung cancers. Patients with deleterious mutations in *PTEN*, including nonsense, frameshift and splicing alterations exhibited a higher progression risk than those without (PFS, HR [95% CI] =2.19 [1.12-4.27], *P*=0.02, **Figure 2C**). Similarly, no OS difference was found comparing patients with and without *PTEN* alterations (HR [95% CI] =1.57 [0.67-3.68], *P*=0.29, **Figure 2D**), suggesting that both *MET* and *PTEN* alterations might serve as predictive markers of dCRT response. Preclinical studies have suggested that the NOTCH signaling pathway might also promote radiation resistance (22). Mutations in *NOTCH2* were found to correlate with unfavorable survival outcome (PFS, HR [95% CI] = 2.0 [1.12-3.57], *P*=0.02; OS, HR [95% CI] = 3.12 [1.65-5.89], *P*=0.0002, **Figures 2E, F**).

**Figure 3:**
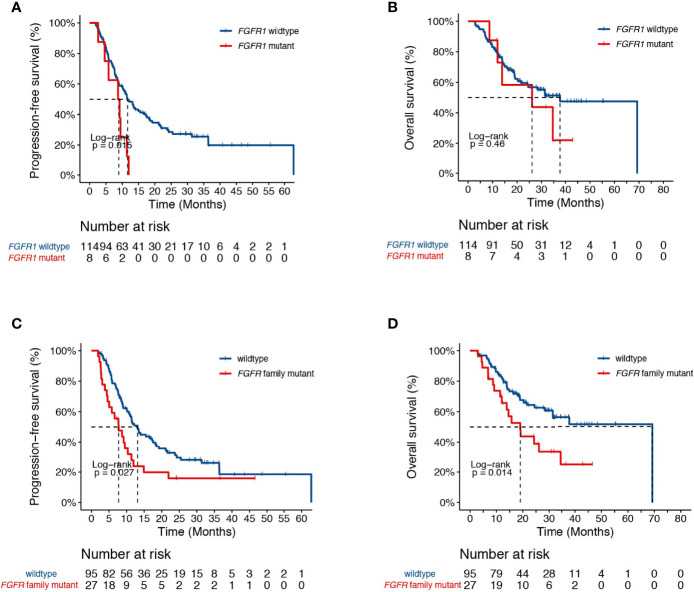
Associations of dCRT survival outcomes with the FGFR pathway. **(A, B)** Kaplan-Meier estimates of **(A)** PFS and **(B)** OS in the full analysis set comparing patients with and without *FGFR1* alterations. **(C, D)** Kaplan-Meier estimates of **(C)** PFS and **(D)** OS in the full analysis set comparing patients with and without alterations in the *FGFR* family genes.

Genetic analysis also revealed alterations in *FGFR1* in association with higher risk of progression in our study cohort (HR [95% CI] = 2.44 [1.16-5.14], *P*=0.015, **Figure 3A**). No difference in survival was observed between *FGFR1* wildtype and mutant patients (HR [95% CI] = 1.41 [0.56-3.56], *P*=0.46, **Figure 3B**). Given that FGFR signaling is often dysregulated in NSCLC and have been implicated in radiation resistance in preclinical studies (23, 24), we sought to further test the association between FGFR family genes and patient survival. Indeed, we found that genetic alterations in the FGFR family receptors, including *FGFR1*-*4*, were associated with earlier progression (PFS, HR [95% CI] = 1.72 [1.06-2.79], *P*=0.03; OS, HR [95% CI] = 2.04 [1.14-3.65], *P*=0.01, **Figures 3C, D**).

Based on these findings, we also assessed if other oncogenic mutations might influence patient outcome. Overall, the presence of oncogenic driver mutations in key lung cancer targets, such as activating mutations in *EGFR*, *ERBB2*, *KRAS*, *MET*, as well as *ALK*, *RET*, and *ROS1* fusions, had no impact on patient progression (HR [95% CI] =0.87 [0.55-1.38], *P*=0.56, **Supplementary Figure 4A**). Interestingly, carriers of oncogenic mutations had significantly improved OS compared with those without (HR [95% CI] =0.52 [0.27-0.99], *P*=0.04, **Supplementary Figure 4B**). Similarly, patients with activating *EGFR* mutations alone had no significant impact on PFS but demonstrated increased OS compared with *EGFR* wild-type patients (PFS, HR [95% CI] =0.77 [0.46-1.26], *P*=0.3; OS, HR [95% CI] =0.42 [0.20-0.89], *P*=0.02, **Supplementary Figures 4C, D**), which is likely explained by the potential use of subsequent targeted therapies in these patients. We also examined the effect of TMB on patient recurrence, and no significant association was identified.

**Figure 4:**
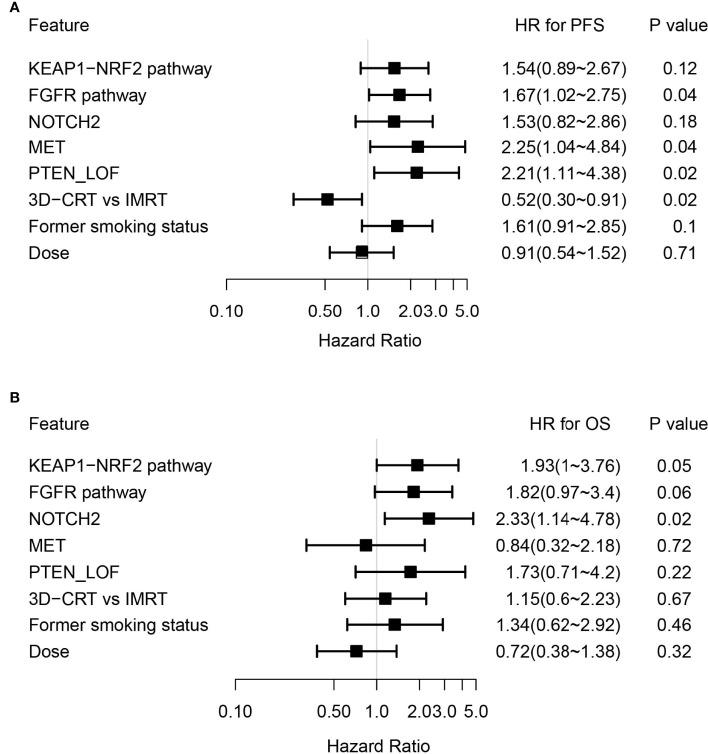
Multivariate Cox analysis of genetic features associated with survival outcomes. **(A, B)** Forest plots showing key genetic and clinical features in association with **(A)** PFS and **(B)** OS following dCRT treatment by multivariate analysis.

**Figure 5:**
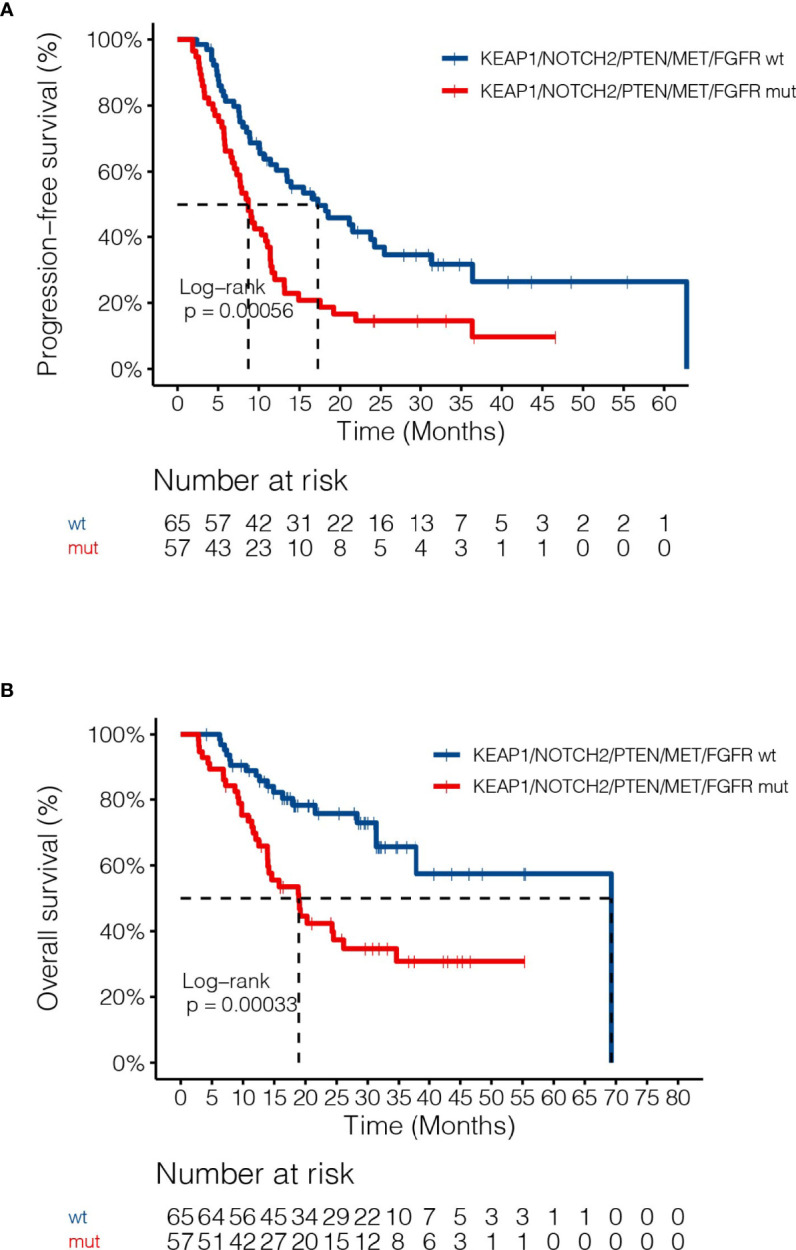
Associations of dCRT survival outcomes with the mutant subgroup. **(A, B)** Kaplan-Meier estimates of **(A)** PFS and **(B)** OS in the full analysis set comparing patients harboring any of the *MET*, *NOTCH2* and *PTEN* loss of function, as well as FGFR and KEAP1-NRF2 pathway gene alterations and those without.

**Figure 6:**
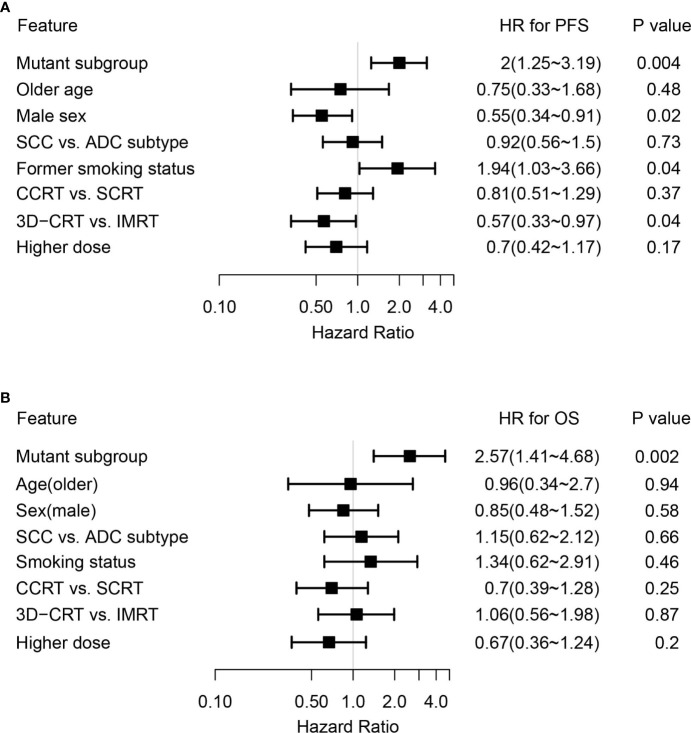
Multivariate Cox analysis of clinical features associated with survival outcomes in the genetically altered subgroup. **(A, B)** Forest plots showing the mutant subgroup and all relevant clinical features in association with **(A)** PFS and **(B)** OS following dCRT treatment by multivariate analysis. Mutant subgroup: patients with any of the *MET*, *NOTCH2* and *PTEN* loss of function, as well as FGFR and KEAP1-NRF2 pathway gene alterations; ADC: adenocarcinoma; SCC: squamous cell carcinoma.

By adjusting for differences in clinical characteristics, including types of RT, smoking histories and overall dose, multivariate Cox analysis showed that alterations in *MET* and deleterious mutations in *PTEN*, as well as the FGFR pathway gene alterations remained independent predictive factors for reduced PFS following CRT (**Figure 4A**). On the other hand, the associations of OS with alterations in *NOTCH2* and those in genes in the FGFR and the KEAP1-NRF2 pathways remained independent (**Figure 4B**). Subgroup analysis considering all patients who had received the recommended doses of 60-66Gy was performed and we found that deleterious mutations in *PTEN* and *FGFR1* mutations remained independently associated with poorer PFS (**Supplementary Table 2**). In addition, mutations in *NOTCH2* remained independent predictors of unfavorable OS outcome (**Supplementary Table 2**). Building on these associations and the largely exclusive nature of *KEAP1*, *MET*, *PTEN*, *NOTCH2* and *FGFR* family gene alterations, we next sought to improve the stratification of NSCLC patients with differential survival outcome. Combined analysis of the mutant subgroup showed markedly improved risk stratification of our patients with significant PFS (HR=2.09, 95% CI=1.36-3.2, *P*=0.0006) and OS (HR= 2.73, 95% CI=1.54-4.85, *P*=0.0003) differences (**Figures 5A, B**). In addition, multivariate analysis incorporating all relevant clinical characteristics, including histology, modes of CRT, smoking status and overall dose, showed that the mutant subgroup remained a strong independent predictor of survival outcomes (**Figures 6A, B**).

### SNPs and Somatic Mutations Predictive of Radiation Toxicity

**Figure 7:**
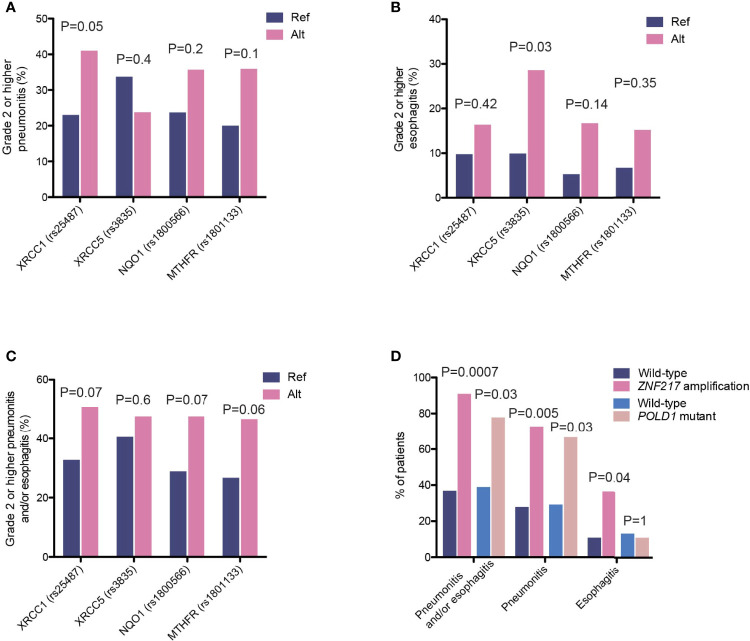
Genetic variants associated with incidence of high-grade radiation toxicity. **(A–C)** The proportions of patients carrying the indicated polymorphisms that developed high-grade **(A)** radiation-induced pneumonitis, **(B)** radiation-induced esophagitis, and **(C)** overall pneumonitis and esophagitis. **(D)** The proportions of patients carrying *ZNF217* amplification or *POLD1* mutations that developed high-grade radiation-induced toxicity events as indicated.

To identify potential risk factors that could explain individual variations in their likelihood to develop radiotherapy-associated toxicity, we first examined the potential effects of various clinical characteristics and treatment regimens. None of the clinical features, including age, smoking status, dose, sequential or concurrent combinations of chemo- and radiotherapy, or delivery methods, had a significant influence on the development of radiation-induced thoracic toxicity, including grade 2 or higher pneumonitis and esophagitis. In recognition that radiotherapy-associated toxicity are manifested as damages to the normal tissue surrounding the site of lesion, we analyzed the association between SNPs and the incidence of radiation-induced thoracic toxicity. Consistent with existing studies demonstrating the associations of radiotherapy-associated toxicity with SNPs in genes in the DNA damage repair, oxidative reduction and metabolic pathways, we identified SNPs in X-ray repair cross-complementing 1 (*XRCC1*, rs25487, c.1196A>G; OR=2.31 [95%CI, 1.0-5.56]; *P*=0.05) and *XRCC5* (rs3835, c.2110-2408G>A; OR=3.59 [95% CI, 0.93-12.96]; *P*=0.03), which conferred increased risks of radiation-induced pneumonitis and esophagitis, respectively (**Figures 7A, B**). Further analysis revealed a stronger association between the *XRCC5* allele with severe (grade 3 or higher) esophagitis (OR=5.71 [95% CI, 1.30-25.0]; *P*=0.03). In addition to these two SNPs, *MTHFR* (rs1801133, c.665C>T) and NAD(P)H Quinone Dehydrogenase 1 (*NQO1*, rs1800566, c.559C>T) were associated with trends towards higher incidence of radiotherapy-associated toxicity overall (**Figure 7C**).

We also observed enrichments of several somatic aberrations in patients who developed grade 2 or higher pneumonitis and/or esophagitis (**Figure 7D**). The zinc-finger protein 217 (*ZNF217*) gene is frequently amplified in human cancers (25, 26). It encodes a Kruppel-like transcription factor that mediates complex molecular processes through the regulation of gene expression. A total of 11 patients carried *ZNF217* amplifications, ten of which developed grade 2 or higher pneumonitis and esophagitis (90.9% vs. 36.9%, OR=16.7 [95% CI, 2.24-748.4], *P*=0.0007, **Figure 7D**). Of these, four developed radiation-induced esophagitis (36.4% vs. 10.8%, OR=4.6 [95% CI, 0.86-21.7], *P*=0.04) and eight developed pneumonitis (72.7% vs. 27.9%, OR=6.8 [95% CI, 1.5-42.1], *P*=0.005). Similarly, *ZNF217* amplification was associated with severe pneumonitis and esophagitis (grade 3 or higher; OR=7.5 [95% CI, 2.0-28.0], *P*=0.003), as well as severe radiation-induced pneumonitis (OR=4.9 [95% CI, 1.3-18.2], *P*=0.02). In addition, we found that mutations in *POLD1*, encoding the DNA polymerase delta 1 that is a key protein for ensuring the replicative fidelity of DNA, were also associated with an increase in overall toxicity risk (pneumonitis and/or esophagitis, 77.8% vs. 38.9%, OR=5.4 [95% CI, 0.97-55.7], *P*=0.03, **Figure 7D**). Six (out of nine) *POLD1* mutations were predicted to have functional consequences (SIFT score ≤ 0.01). Of the seven patients who developed toxicity, six had pneumonitis and one had esophagitis. It is worth noting that somatic alterations in both genes showed stronger associations with radiation pneumonitis, as compared to esophagitis, likely reflecting interactions of these tumor cells with the local microenvironment.

## Discussion

As we enter the era of personalized medicine, there is an area of unmet needs for identifying genetic determinants that explain individual differences in dCRT response. The development of disease recurrence and severe radiation-induced toxicity following dCRT could negatively impact patients’ survival outcome and quality of life. In this study, by comprehensive profiling of 122 unresectable stage IIIA-C NSCLC patients who underwent dCRT, we identified a number of highly relevant and novel genetic and pathway-level features that might serve as potential biomarkers for predicting response to CRT.

The KEAP1-NRF2 pathway is often altered in NSCLC. In line with previous studies that have demonstrated the potential prognostic or predictive value of the dysregulation in the KEAP1-NRF2 pathway in NSCLC patients following chemotherapy and/or radiotherapy (20, 21, 27, 28), our findings independently confirmed its association with poor outcome in NSCLC patients following dCRT. Multiple others signaling pathways, including the MAPK, PI3K/AKT, FGFR and NOTCH pathways, have been implicated in radio-resistance, through their regulation of cellular proliferation, differentiation, apoptosis, invasion and maintenance of cancer stem cells (22, 23, 29–32). However, most findings were based on preclinical studies using *in vitro* or animal models. Here, we provided the first clinical evidence showing the associations of dCRT recurrence with several highly functionally relevant lung cancer genes, including *MET*, *PTEN*, *FGFR1*-*4*, and *NOTCH2*. Of these, *MET*, *PTEN* and *FGFR*s remained independent predictors of PFS by multivariate analysis. In particular, MET is commonly activated in NSCLC, which can be a result of gene amplification and exon 14 skipping mutations. In our study, the majority of *MET*-altered cases could lead to activation of the protein, which included *MET* exon 14 skipping mutations, gene rearrangement and amplification. While the presence of *MET* alterations was associated with worse outcome when treated with dCRT, no difference in OS was detected. Despite insufficient clinical information on the subsequent lines of treatment, these *MET*-altered cases might have derived long-term benefit from MET-targeted therapies. Similarly, while the presence of other potentially targetable driver mutations, such as *EGFR* activating mutations, had no impact on disease progression, it was associated with improved OS, which is also likely explained by the later use of targeted therapies in these patients. Thus, our data suggest that *MET* alteration is likely a negative predictive, rather than prognostic, factor of dCRT recurrence.

The FGFR family receptors activate multiple signaling pathways, including the RAS/MAPK, PI3K, and STAT pathways, which play important roles in cancer initiation and development (33). Emerging evidence suggest that FGFR may be implicated in variable response to radiotherapy. Pre-clinical studies in NSCLC cell lines, xenograft models and genetically engineered mouse models have shown that FGFR inhibition can enhance radiation response, which may be through the upregulation of cellular apoptosis and autophagy (23) and/or polarization of tumor-associated macrophages towards the M1 phenotype (24). In our study, we provide the first clinical evidence for the role of FGFR in mediating dCRT response. Patients with FGFR family gene alterations demonstrated reduced PFS outcome. Furthermore, the negative association of FGFRs with PFS also translated into poor OS outcome.

In addition to disease recurrence, a subset of patients would also develop severe, and often long-term, radiation-induced toxicities, which can negatively impact their quality of life. Understanding the genetic basis underlying individual differences in the development of dCRT-associated adverse events would allow for the risk stratification of patients and consequently personalized dCRT regimens, which would maximize tumor control while minimizing damage to the local tissue. The involvement of SNPs in the various damage and stress-response genes in mediating radiation-induced toxicities has been extensively studied in multiple cancer types (9–12, 34–37). Early association studies have employed a candidate gene approach, which has led to the identification of several key genes that may serve as potential predictors of radiation-induced toxicities, including *ATM*, base excision repair genes (*XRCC1*-*5*), mismatch repair genes (*MSH2*, *MLH1*), and oxidative damage-detoxification genes (*GSTM1*, *GSTT1*) (38–45). As we shift from candidate gene approach to genome-wide association studies, such as the multi-centered RAPPER (Radiogenomics: Assessment of Polymorphisms for Predicting the Effects of Radiotherapy) study (46), more genetic polymorphisms associated with radio-toxicity have been identified. However, these studies are often underpowered and difficult to replicate due to the small effect size of individual SNPs on radiotoxicity, and rarely lead to clinically useful biomarkers. A gene-expression-based radio-sensitivity model has been developed (47–50) and clinically validated in multiple cancer types (51–55). These data provide the prescription framework for genomic-based radiotherapy and emphasize the importance of multi-gene testing as response to CRT is dependent on the combined influence of genetic variations at multiple loci. However, it remains challenging to adopt RNA-sequencing routinely into clinical practice, particularly on formalin-fixed paraffin-embedded samples.

Taking advantage of the NGS technology, our study independently verified the predictive potential of the DNA damage repair and oxidative stress pathway gene variants for radiotherapy-associated toxicity. Specifically, we identified polymorphisms in *XRCC1*/*XRCC5* (x-ray repair cross-complementing 1/5), encoding two key genes responsible for base excision repair, that were associated with differential risks of high-grade toxicity. The *XRCC1* rs25487 allele has been associated with severe oral mucositis in oropharyngeal carcinoma patients treated with radiotherapy (45). The *XRCC5* rs3835 allele has been implicated in the development of severe radiation pneumonitis in NSCLC patients (56). In our study, no significant association between this particular allele with radiation pneumonitis was observed. Instead, we showed that *XRCC5* might increase the risk of developing severe radiation esophagitis. In addition, we identified two risk alleles in *MTHFR* and *NQO1* that were associated with radiotoxicity. *MTHFR* encodes a methylenetetrahydrofolate reductase, which participates in folate metabolism and the regulation of DNA methylation and repair (57, 58). On the other hand, NQO1 is involved in the regulation of reactive oxygen species and continued oxidative stress can also induce DNA damage and chronic inflammation (59, 60). Combinatorial testing for these genetic variations might be useful for identifying patients who are susceptible to radiation toxicity. However, large-scale studies are needed to fully assess the predictive potential of these particular polymorphisms or variations in DNA damage repair and oxidative stress pathways.

Solid tumors often exhibit complex interactions with their surrounding tissues *via* stromal components, the vasculature and immune cells, among others (61). However, it has never been reported that somatic mutations could influence a patient’s likelihood of developing radiation toxicity. Here, we report the associations of *ZNF217* amplification and *POLD1* mutations with increased likelihoods of developing radiation toxicity. In particular, *ZNF217* amplification was associated increased risks of developing severe (grade 3 or higher) pneumonitis and esophagitis. *ZNF217* is commonly amplified in human cancers (25, 26). While the presence of *ZNF217* amplification itself may indicate loss of genome integrity, there may also be functional consequences given that *ZNF217* encodes a transcription factor that mediates a diverse array of cellular processes through the regulation of various target gene expressions (62). Importantly, ZNF217 may have a role in DNA damage repair as it has been shown to repress the levels of BRCA1 (63). Likewise, the role of POLD1 in controlling replicative fidelity has been firmly established (64, 65). Thus, we speculate that *ZNF217* amplification and/or *POLD1* mutations in the tumor may affect overall genome stability and lead to the generation of tumor-specific neoantigens and consequently extensive lymphocyte infiltration (66). Conceivably, this could also exacerbate inflammation in the surrounding normal tissue following radiation. Interestingly, similar to our work in small-cell lung cancer (67), somatic alterations are more likely to affect radiotherapy-associated toxicity at the site of the lesion, as they were more commonly associated with pneumonitis than with esophagitis. Due to the retrospective nature of this current study, our cohort consisted of relatively high proportions of SCC and former smokers, as well as widely varied radiation doses which is associated with radiotherapy toxicity (68). Thus, the predictive value of these novel variations’ merits further investigation. Nevertheless, our data indicate that extra caution should be exercised when giving radiotherapy to NSCLC patients carrying such mutations.

Our observations from clinical data of genetic associations with CRT survival outcome and toxicity provide a set of candidate predictive biomarkers present in normal and also tumor tissues. The mechanisms by which some of these genetic variants act to promote development of adverse response or cancer recurrence remain to be elucidated, although it is likely through their combined influence on important oncogenic signaling pathways, as well as DNA damage repair, oxidative and inflammatory response pathways. Due to the lack of sufficient data from published work or public databases such as TCGA, future work should involve validation of these potential biomarkers in a larger set of cohorts and generation of multifactorial prediction models of the expected treatment outcome. Taken together, our results demonstrate the clinical utility of NGS panels in identifying predictive biomarkers for response to CRT and suggest that testing for these susceptibility loci would prove beneficial in improving personalized CRT in NSCLC patients.

## Data Availability Statement

The original contributions presented in the study are publicly available. This data can be found here: Genome Sequence Archive for Human (GSA- Human); HRA002626.

## Ethics Statement

The studies involving human participants were reviewed and approved by Ethical Review Board of the Oncology Center of Shandong Provincial Hospital. The patients/participants provided their written informed consent to participate in this study.

## Author Contributions

Conceptualization: JY, SY, YS, XW, and XNW. Investigation: JY, SY, YS. Resources: DM, HG, JW, JM, ZY, XZ, LD, PC. Data curation: KH, ST, LL. Data analysis and interpretation: JP, JCY, JZ, DM and JM. Manuscript writing: All authors. Manuscript review & editing: All authors. Funding acquisition: JY. Supervision: JY, SY, YS. All authors contributed to the article and approved the submitted version.

## Funding

This study was supported by Natural Science Foundation of China (grant No. NSFC81872475 and NSFC82073345 to SY; NSFC81874254 to PC); and Research Unit of Radiation Oncology, Chinese Academy of Medical Sciences (2019RU071), the foundation of National Natural Science Foundation of China (NSFC81627901, NSFC81972863 and NSFC82030082), the foundation of Natural Science Foundation of Shandong (ZR201911040452) and the Academic Promotion Program of Shandong First Medical University (2019ZL002) to JY.

## Conflict of Interest

JP, JY, JZ, HB, XW, XNW and YS are staff of Nanjing Geneseeq Technology Inc.

The remaining authors declare that the research was conducted in the absence of any commercial or financial relationships that could be construed as a potential conflict of interest.

## Publisher’s Note

All claims expressed in this article are solely those of the authors and do not necessarily represent those of their affiliated organizations, or those of the publisher, the editors and the reviewers. Any product that may be evaluated in this article, or claim that may be made by its manufacturer, is not guaranteed or endorsed by the publisher.
