# Supplementary material for: Genomic Profiling Reveals Novel Predictive Biomarkers for Chemo-Radiotherapy Efficacy and Thoracic Toxicity in Non-Small-Cell Lung Cancer
Source: Front Oncol. 2022 Jul 14;12:928605. doi: 10.3389/fonc.2022.928605 (PMC9329611; doi:10.3389/fonc.2022.928605)
Supplement: Supplementary file 1 [file Image_1.pdf]

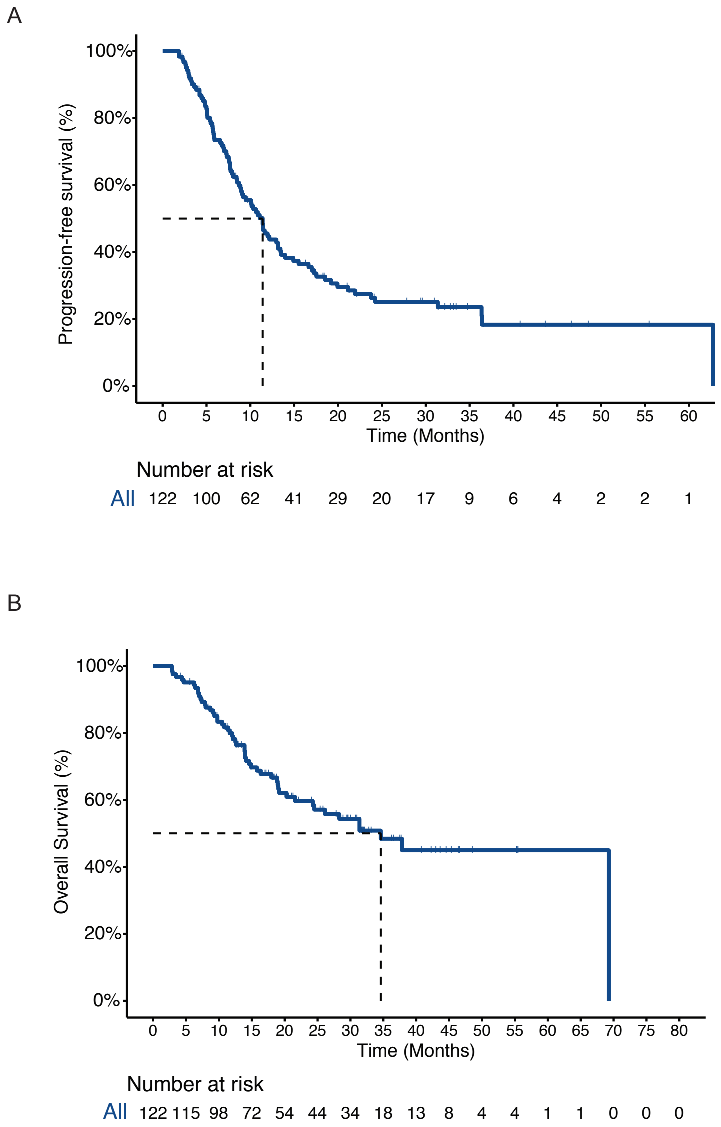

Supplementary Figure 1. Survival outcomes of the entire cohort. (A and B) Kaplan-Meier estimates of (A) PFS and (B) OS in the full analysis.
