# Supplementary material for: Genomic Profiling Reveals Novel Predictive Biomarkers for Chemo-Radiotherapy Efficacy and Thoracic Toxicity in Non-Small-Cell Lung Cancer
Source: Front Oncol. 2022 Jul 14;12:928605. doi: 10.3389/fonc.2022.928605 (PMC9329611; doi:10.3389/fonc.2022.928605)
Supplement: Supplementary file 2 [file Image_2.pdf]

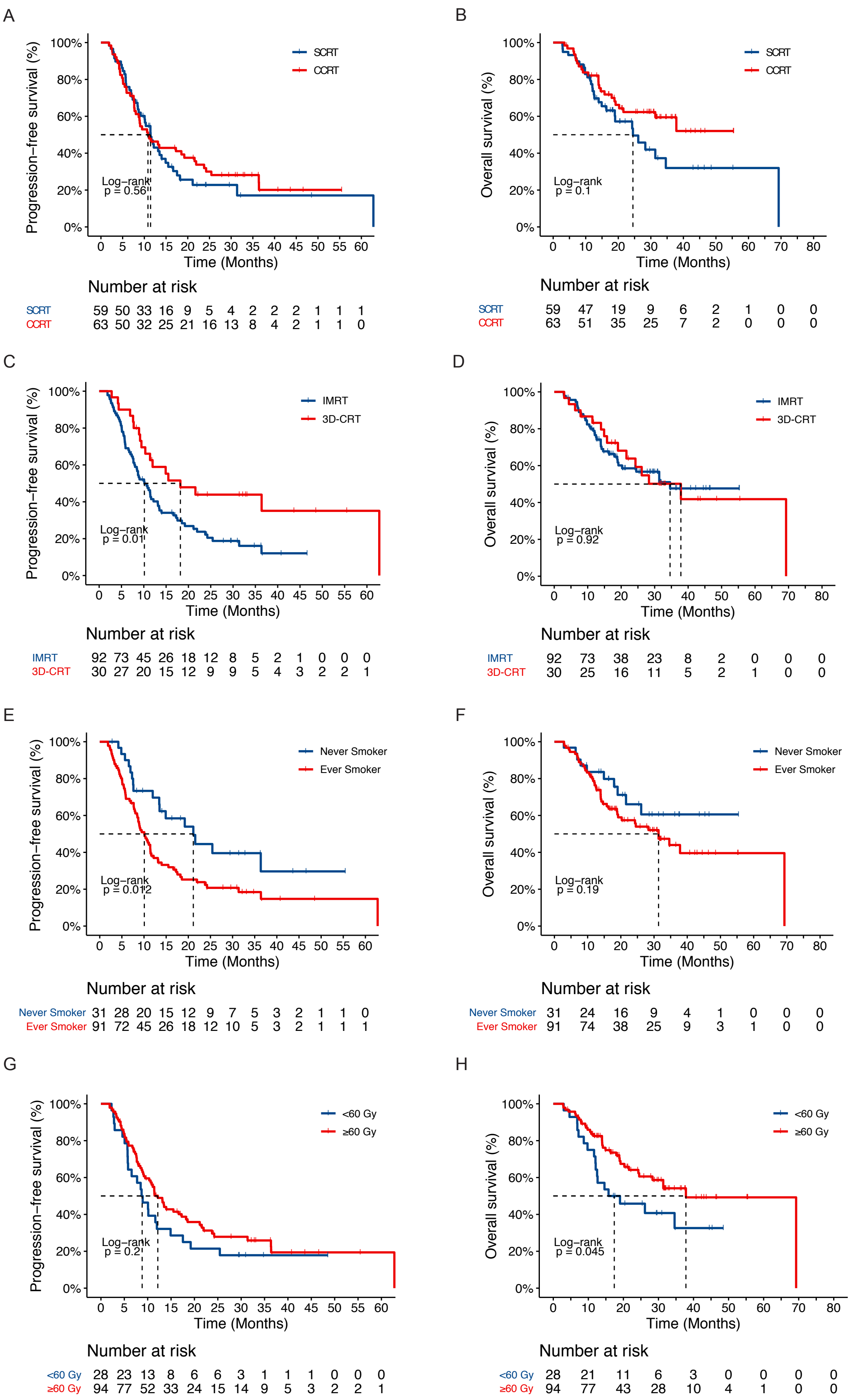
A
Progression−free survival (%)
SCRT
CCRT
Log−rank
p = 0.56
Time (Months)
Number at risk
SCRT 59 50 33 16 9 5 4 2 2 2 1 1 1
CCRT 63 50 32 25 21 16 13 8 4 2 1 1 0
B
Overall survival (%)
SCRT
CCRT
Log−rank
p = 0.1
Time (Months)
Number at risk
SCRT 59 47 19 9 6 2 1 0 0
CCRT 63 51 35 25 7 2 0 0 0
C
Progression−free survival (%)
IMRT
3D-CRT
Log−rank
p = 0.01
Time (Months)
Number at risk
IMRT 92 73 45 26 18 12 8 5 2 1 0 0 0
3D-CRT 30 27 20 15 12 9 9 5 4 3 2 2 1
D
Overall survival (%)
IMRT
3D-CRT
Log−rank
p = 0.92
Time (Months)
Number at risk
IMRT 92 73 38 23 8 2 0 0 0
3D-CRT 30 25 16 11 5 2 1 0 0
E
Progression−free survival (%)
Never Smoker
Ever Smoker
Log−rank
p = 0.012
Time (Months)
Number at risk
Never Smoker 31 28 20 15 12 9 7 5 3 2 1 1 0
Ever Smoker 91 72 45 26 18 12 10 5 3 2 1 1 1
F
Overall survival (%)
Never Smoker
Ever Smoker
Log−rank
p = 0.19
Time (Months)
Number at risk
Never Smoker 31 24 16 9 4 1 0 0 0
Ever Smoker 91 74 38 25 9 3 1 0 0
G
Progression−free survival (%)
<60 Gy
≥60 Gy
Log−rank
p = 0.2
Time (Months)
Number at risk
<60 Gy 28 23 13 8 6 6 3 1 1 1 0 0 0
≥60 Gy 94 77 52 33 24 15 14 9 5 3 2 2 1
H
Overall survival (%)
<60 Gy
≥60 Gy
Log−rank
p = 0.045
Time (Months)
Number at risk
<60 Gy 28 21 11 6 3 0 0 0 0
≥60 Gy 94 77 43 28 10 4 1 0 0

Supplementary Figure 2. Clinical correlates of dCRT survival in NSCLC. (A and B) Kaplan-Meier estimates of (A) PFS and (B) OS in the full analysis set comparing patients treated with concurrent vs. sequential CRT. (C and D) Kaplan-Meier estimates of (C) PFS and (D) OS in the full analysis set comparing patients treated with 3D-CRT vs. IMRT. (E and F) Kaplan-Meier estimates of (E) PFS and (F) OS in the full analysis set comparing patients with and without smoking histories. (G and H) Kaplan-Meier estimates of (G) PFS and (H) OS in the full analysis set comparing patients treated with different RT doses. HR, hazard ratio; CI, confidence interval. Tick marks indicate censored data.
