# Supplementary material for: Genomic Profiling Reveals Novel Predictive Biomarkers for Chemo-Radiotherapy Efficacy and Thoracic Toxicity in Non-Small-Cell Lung Cancer
Source: Front Oncol. 2022 Jul 14;12:928605. doi: 10.3389/fonc.2022.928605 (PMC9329611; doi:10.3389/fonc.2022.928605)
Supplement: Supplementary file 4 [file Image_4.pdf]

Supplementary figure S4

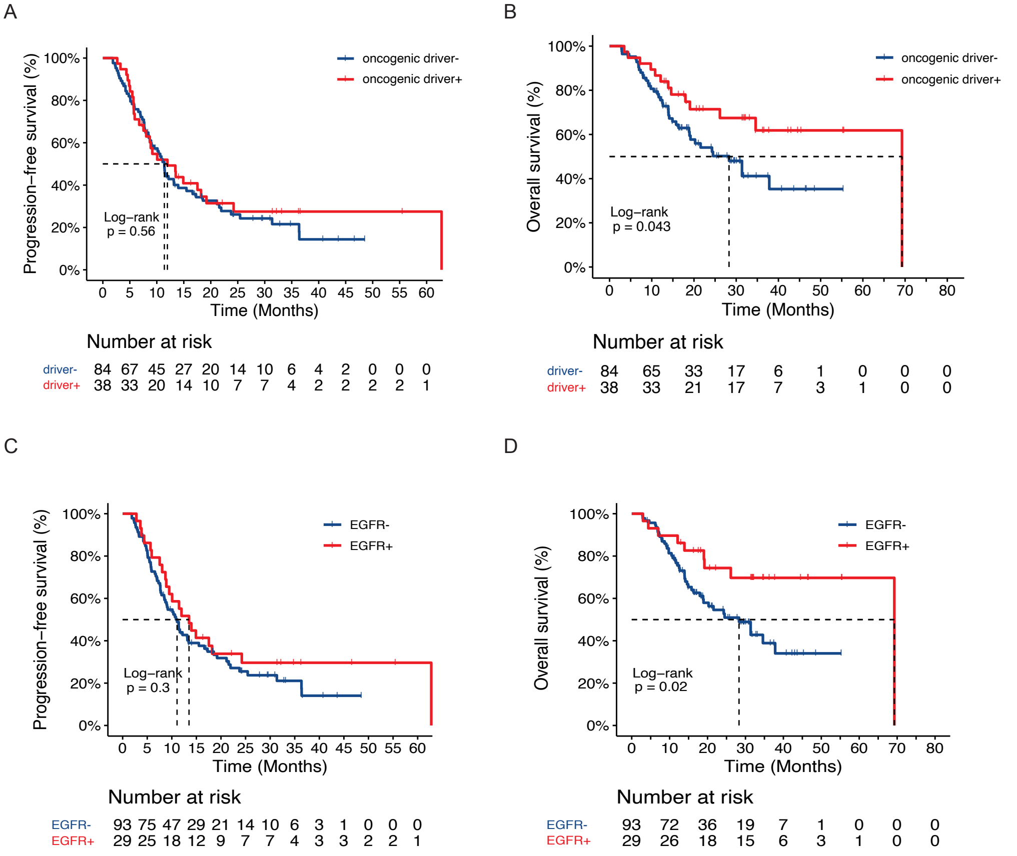

Figure S4. Associations of dCRT recurrence with oncogenic driver mutations.

(A, B) Kaplan-Meier estimates of (A) PFS and (B) OS in the full analysis set comparing patients with and without oncogenic driver mutations. (C, D) Kaplan-Meier estimates of (C) PFS and (D) OS in the full analysis set comparing patients with and without *EGFR* mutations.
